# The Anterior Insula Processes a Time-Resolved Subjective Risk Prediction Error

**DOI:** 10.1523/JNEUROSCI.2302-24.2025

**Published:** 2025-04-23

**Authors:** Jae-Chang Kim, Lydia Hellrung, Stephan Nebe, Philippe N. Tobler

**Affiliations:** ^1^Zurich Center for Neuroeconomics, Department of Economics, University of Zurich, Zurich 8006, Switzerland; ^2^Neuroscience Center Zurich, University of Zurich, Swiss Federal Institute of Technology Zurich, Zurich 8057, Switzerland; ^3^University Research Priority Program “Adaptive Brain Circuits in Development and Learning” (URPP AdaBD), University of Zurich, Zurich 8057, Switzerland

**Keywords:** attention, learning, uncertainty

## Abstract

The insula processes errors in the prediction of risky, motivationally relevant outcomes and thereby is thought to respond similarly to better-than-predicted and worse-than-predicted outcomes. However, the nature of the encoded risk prediction error signals remained unclear. Moreover, the insula was proposed to preferentially process events and stimuli in the aversive domain, rather than in a domain-general fashion. Here, we aimed to illuminate these issues. In a Pavlovian task, participants (*n* = 41; 19 women) rated both cues and outcomes, allowing us to quantify not only objective but also trial-specific subjective risk prediction errors. We found preferential coding of subjective risk prediction errors in the anterior insula and adjacent frontal cortex. This contrasted with preferential coding of objective risk prediction errors in the mid-insula. The anterior insula encoded the subjective risk prediction errors not only at the time of outcomes but also at the time of cues, in line with a temporally fine-grained computation of these prediction errors. Cue-induced subjective risk prediction error signals occurred predominantly in the aversive domain, while outcome-induced subjective risk prediction error signals occurred also in the appetitive domain. Domain-specific analyses of risk prediction errors elicited by the preceding outcome at the time of the next cue indicated that the anterior insula updates risk predictions more strongly in the aversive than the appetitive domain. Together, our findings specify the nature of risk prediction errors processed by the anterior insula as subjective, time-resolved, partly domain-general (outcome), and partly domain-preferential (cue), thereby reconciling apparently disparate lines of research.

## Significance Statement

The anterior insula is a hub of the salience network, thought to process both good and bad surprises. However, salience needs defining, and the nature of salience signals in anterior insula remained unclear. Here, we define salience as risk and use a Pavlovian task with subjectively evaluated cues and liquid outcomes, allowing us to determine unsigned (i.e., risk) prediction errors, at both time points. In a double dissociation, subjective risk prediction error signals occurred preferentially in anterior insula and objective signals in mid-insula. We also show that subjective risk prediction errors are preferentially encoded for aversive rather than appetitive cues. These findings unify divergent frameworks of insula function, specifying the insula's nuanced role in salience processing for motivated behavior.

## Introduction

The insula has been associated with at first sight mutually exclusive behavioral functions. In keeping with the insula being a convergence hub of diverse inputs (Kwon et al., 2025), it has been proposed to play a pivotal role in determining salience for goal-directed action (Zhang et al., 2017; Molnar-Szakacs and Uddin, 2022). Supporting a role in motivational salience processing, particularly the anterior part of the insula encodes surprising valenced outcomes, regardless of whether they are appetitive or aversive (Rutledge et al., 2010; Metereau and Dreher, 2013; Fouragnan et al., 2018; Corlett et al., 2022; Haufler et al., 2022; Horing and Büchel, 2022). In this view, the anterior insula is activated whenever outcomes are better or worse than predicted, i.e., whenever there is an unsigned error in the prediction of appetitive or aversive outcomes. Mathematically, this view defines salience as variance risk (expected squared deviation from the mean; Preuschoff et al., 2008). Yet, the precise nature of the insular risk prediction error signal remains to be determined. For example, it remains unclear whether the insula encodes errors in objective (as determined by the difference between outcomes and expected values of the cues) or subjective (as determined by individual valuation of outcomes and cues) risk.

A different line of research reports preferential insular encoding of aversive rather than, or over and above, appetitive events. For example, direct meta-analytic comparisons find stronger insular encoding of prediction errors in the aversive than the appetitive domain (Corlett et al., 2022). Moreover, the insula processes negative affect induced through thermally, mechanically, auditorily, or visually aversive stimulation rather than pleasantness of appetitive events (Čeko et al., 2022). The insula also encodes risk (Preuschoff et al., 2008) and relative loss (Kuhnen and Knutson, 2005), which for most decision-makers are aversive and traded off against expected reward. While they differ on the functional forms (e.g., concave utility function vs negatively evaluated mean-preserving spread), both economic (Von Neumann and Morgenstern, 1944; Bernoulli, 1954) and finance theories (Markowitz, 1959) converge on risk being typically aversive, at least in the gain domain (Kahneman and Tversky, 1979). Empirical findings concur (Holt and Laury, 2014; Burke et al., 2018; Kim et al., 2025) and suggest that during anticipation of high-risk gambles, insula activity is particularly increased in risk-averse individuals (Rudorf et al., 2012). In contrast to risk, the expected reward is appetitive and preferentially associated with striatum rather than insula activity (Bossaerts, 2010). Although risk processing as such is also compatible with salience coding as mentioned above, in this view, the activation of the insula would arise primarily from the aversive nature of risk. More generally, the insula would primarily encode worse-than-predicted outcomes and their affective consequences.

**Figure 1.**
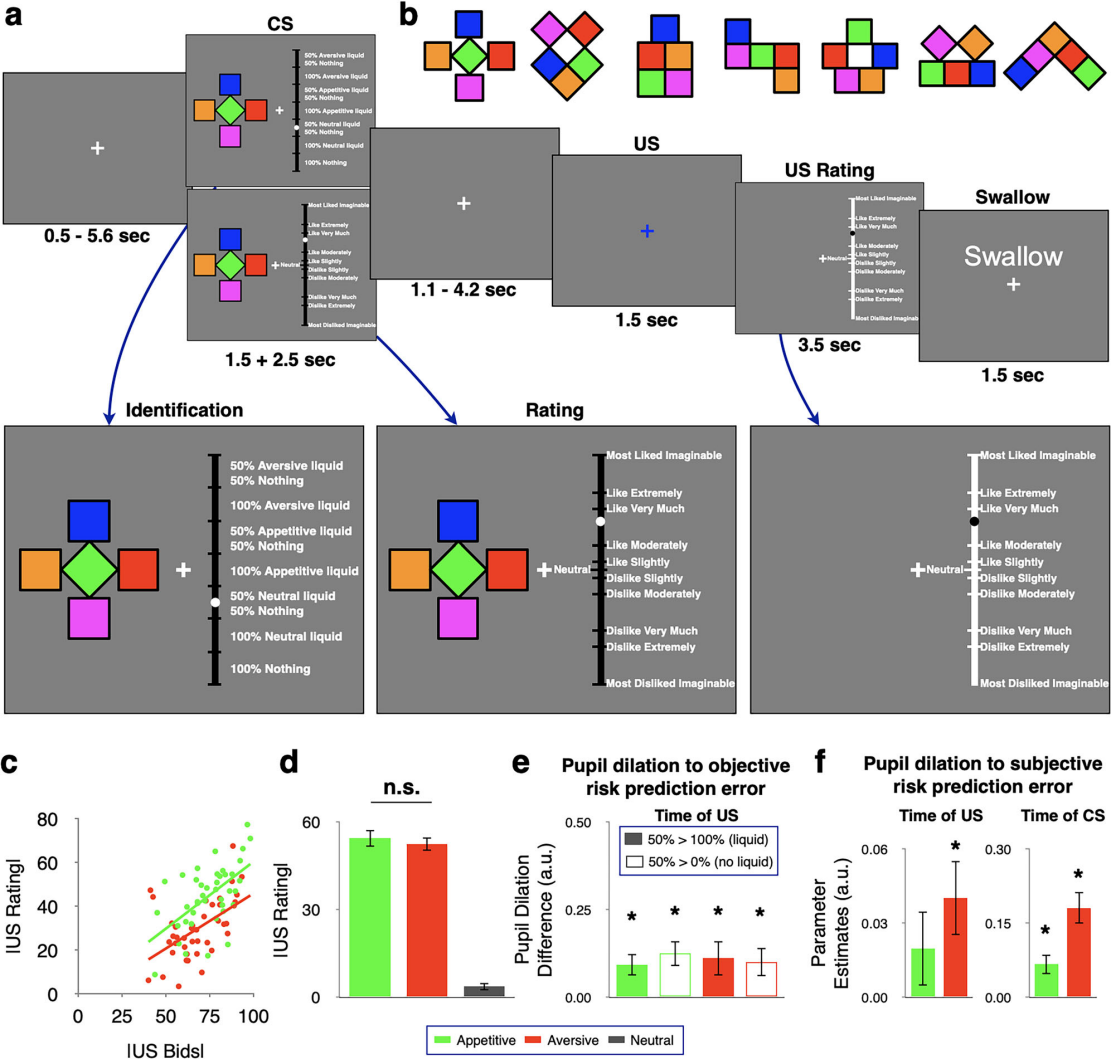
Experimental design, absolute value of liquids, and pupil diameter. ***a***, Example trial from the main task. Each trial presented one prelearned visual cue predicting one of three types of liquids, i.e., appetitive, aversive, and neutral (*p* = 0.5, 1), or no outcome (*p* = 0). Participants either indicated the associated outcome or rated cue pleasantness, with 1.5 s to view the cue and an additional 2.5 s to respond. A variable delay (1.1–4.2 s) preceded the outcome, which was marked by the fixation cross turning blue, and followed by an outcome pleasantness rating. Finally, participants rinsed with the neutral liquid and were instructed to swallow. Each participant completed four runs per day (2 d in total), with each run comprising 56 trials. ***b***, The seven cues used in the task were different arrangements of the same building blocks. The cue–to–condition assignment was counterbalanced across participants. ***c***, Absolute ratings and absolute bids of liquids showed a significant and similar positive correlation in the appetitive (*r* = 0.58, *p* < 0.001) and aversive (*r* = 0.54, *p* < 0.001) domains. Responses to the two liquids of each domain were averaged. ***d***, Absolute rating (a measure of subjective salience) of liquids used in the task. Ratings of liquids delivered in a deterministic fashion (*p* = 1) were averaged across all individuals, revealing no significant difference between appetitive (green) and aversive (red) liquids but lower absolute values for neutral liquid. ***e***, Objective risk prediction errors increase pupil size. Pupil dilation was greater for outcomes eliciting objective risk prediction errors (following *p* = 0.5 cues) than for outcomes not eliciting objective risk prediction errors (following *p* = 0 or *p* = 1 cues). These effects occurred both in the appetitive (green) and aversive (red) domains, regardless of whether liquids were delivered (filled bars) or not (empty bars). ***f***, Outcome- and cue-induced pupil responses correlate with subjective risk prediction errors. Subjective risk prediction errors at the outcome were defined as absolute difference between outcome and cue rating. Similarly, subjective risk prediction errors induced by cues were calculated as absolute difference between the rating of the presented cue and the participant- and block-specific average rating of all cues. Error bars indicate ±1 standard error of the mean. Abbreviations: CS, conditioned stimulus; US, unconditioned stimulus.

The two views are not mutually exclusive, as the insula could both encode an unsigned prediction error and do so more strongly in the aversive than the appetitive domain on top of that. With this reasoning, the question arises as to how exactly the insula achieves this dual role. To address the subjective versus objective nature of unsigned prediction errors and their domain generality in the insula, we used a Pavlovian conditioning task involving appetitive, aversive, and neutral liquids (Fig. 1*a*). We behaviorally matched the absolute value of appetitive and aversive liquids and used different cues to predict the liquids with different probabilities. In each trial, participants rated both the cue and the outcome, which allowed us to determine subjective risk prediction errors as the unsigned difference between the rating of the current stimulus (e.g., outcome) and the rating of the prediction (e.g., cue).

## Materials and Methods

### Participants

After a screening session [see below, screening session and cue learning (Day 1)], 50 healthy, right-handed volunteers (25 women) participated in the functional magnetic resonance imaging (fMRI) sessions of this study. Inclusion criteria were age between 18 and 40 years, near-perfect vision, and nonsmoking. Exclusion criteria were arm or hand injuries, metal implants in the body, large tattoos in the region of the head or neck, claustrophobia, current medication, and neurological, psychiatric, or eating disorders as ascertained by telephone-based screening. Nine participants were excluded from analyses, because they did not correctly identify the meaning of cues (<60% of trials; *n* = 3), showed excessive head motion (*n* = 2), or experienced technical problems during scanning (*n* = 4). Accordingly, we present data from 41 participants (22.4 ± 0.43 years, mean ± SEM; 19 women). The study was approved by the Research Ethics Committee of the Canton of Zurich, and written informed consent was obtained from all participants before the experiment. We have previously used the present data set to ask different questions [assessment of objectively defined generic vs motivational probability and uncertainty signals at the time of the cue (Kim et al., 2024)].

Inside the scanner, participants performed a Pavlovian task in which visual cues were associated with outcomes from the different domains (appetitive, aversive, neutral) and at different probabilities (*p* = 0, 0.5, 1). Participants had learned the meaning of cues already in the screening session. For each cue, they were asked to either indicate the outcome associated with the cue or rate the pleasantness of the presented cue on a general labeled magnitude scale (gLMS). After a variable delay (1.1–4.2 s), the fixation cross turned blue while the participants received either a 0.2 ml drop of liquid or no liquid. Next, participants rated the pleasantness of the outcome using a trackball device. Finally, they were instructed to swallow and rinse with the neutral liquid.

### Study design and liquid evaluation

Participants attended three lab sessions: an initial screening session and two main task sessions inside the MRI scanner, separated by 1–30 d (mean, 9.46 d; SD, 9.09 d). They were instructed to abstain from eating or drinking for at least 3 h prior to each session. During the first session, we individualized the selection of two appetitive (e.g., fruit juice and milk drink) and two aversive (e.g., hypertonic saline and bitter) liquids, based on each participant's preferences. These liquids along with a neutral liquid (distilled water with main ionic components of saliva), which was the same for all participants, were delivered through custom-built polytetrafluoroethylene tubing connected to a mouthpiece. Liquid delivery was controlled by software integrated with a one-way pump system.

Participants evaluated the liquids in a two-step process. First, they tasted a drop and rated it on a gLMS to express their subjective like or dislike. Then, they bid how much effort they were willing to exert to receive or avoid another drop, measured as a percentage of their maximum hand grip force. The actual price for receiving or avoiding another drop was determined randomly (Becker et al., 1964), and participants had to pay this price (i.e., exert the corresponding effort) if their bid was equal to or higher than the actual price. We used the bids to gauge the subjective value of the liquids and selected liquids in the screening session so that bids were matched between appetitive and aversive domains for each participant. If a match could not be found, the participant could not proceed to the fMRI sessions.

In the screening session, participants also learned to associate the visual cues with the probability of receiving a specific liquid, with successful cue–outcome association learning required for progression to the fMRI sessions. In the main task, participants encountered trials that began with the presentation (1.5 s) of one of seven visual cues (cue-to-condition assignment counterbalanced across participants), followed by a pleasantness rating or an outcome prediction (2.5 s). After a random delay ranging from 1.1 to 4.2 s, the fixation cross changed to blue, signaling the delivery of 0.2 ml of liquid reward or no liquid at all. Participants then appraised the pleasantness of the received outcome with a trackball device. Subsequently, they were prompted to swallow and rinse their mouth with a neutral liquid to prepare for the next trial.

Throughout each trial, we recorded physiological data, including eye gaze, heart rate, and respiration, which we used to correct for physiological motion in the imaging analysis. Moreover, we recorded pupil dilation in response to both cues and outcomes, as an autonomous measure of salience (Liao et al., 2016). Participants completed four blocks of 56 trials per session, encountering each cue eight times. To minimize habituation and/or sensitization to the liquids, we alternated between two sets of appetitive and aversive liquid pairs across the blocks, with the order of presentation balanced over consecutive sessions.

### Neuroimaging

#### Data acquisition

Participants were scanned on a Philips Achieva 3 Tesla MRI scanner with a 32-channel receive-only head coil. Using partial volume acquisition, we focused on the insula and ventral brain regions including the ventral prefrontal cortex, striatum, amygdala, and visual cortex. We collected 390 T2*-weighted echoplanar images (EPI) per run [voxel size, 2 × 2 × 2 mm^3^; field of view (FOV), 192 × 192 × 72 mm^3^; slice gap, 0.2 mm; repetition time (TR), 2,100 ms; echo time (TE), 27 ms; flip angle, 90°; matrix, 96 × 94]. Before acquiring functional data, we collected a 2D multislice dual-echo gradient-echo image (TE, 4.6 and 6.9 ms). From this image, we computed a B0 field map, which during postprocessing served to correct for distortions in the EPI data due to inhomogeneity in the magnetic field. Additionally, a single whole-brain EPI volume (60 slices; voxel size, 2 × 2 × 2 mm^3^; FOV, 192 × 192 × 132 mm^3;^ slice gap, 0.2 mm; TR, 3,545 ms; TE, 27 ms; flip angle, 90°; matrix, 96 × 94) was obtained for improved data coregistration. Finally, we collected a T1-weighted high-resolution anatomical image (3D turbo field echo; 170 slices; slice thickness, 1 mm; no gap; in-plane resolution; 1 × 1 mm^2^; FOV, 256 × 256 × 170 mm^3^; TR, 8 ms; TE, 3.7 ms).

Image preprocessing was performed with Statistical Parametric Mapping (SPM12, Wellcome Department of Imaging Neuroscience; Ashburner et al., 2021) and corrected for slice-time nonsimultaneity, motion, and magnetic field inhomogeneity. Coregistration involved aligning the fMRI data with both the whole-brain EPI and the high-resolution anatomical image. The processed images were then standardized to MNI space and spatially smoothed (6 mm full-width at half-maximum). Physiological noise was modeled and corrected for with the following parameters (Kasper et al., 2017): cardiac pulsation (third-order Fourier expansions), respiration (fourth-order Fourier expansions), and cardiorespiratory interactions (first-order Fourier expansions). To remove spikes, we used the censoring method (Power et al., 2012) with a threshold of 0.6. If >100 volumes were censored within a block (25.6% of the total volumes), the two blocks containing the same pair of liquids were excluded from the study so that all pairs of liquids entered the analyses with the same number of blocks. Participants were excluded from the study if this process resulted in <4 usable blocks (*n* = 2).

### Definition of objective and subjective risk/salience prediction errors

Salience is a multifaceted concept, in need of specification, which we achieve by defining salience mathematically as risk. Here, we also embed salience in the wider landscape and distinguish it from related concepts. Salience captures attention according to the overall importance of the stimulus (Kahnt and Tobler, 2017). The importance of a stimulus can be driven by its physical and sensory properties (Lauffs et al., 2020), in comparison with other stimuli (Modinos et al., 2020), or by the motivating (appetitive or aversive) nature of the consequences it predicts (Kahnt and Tobler, 2013). Motivational salience is an unsigned currency (Haufler et al., 2022); both negative and positive outcomes increase the salience of a stimulus predicting them and the attention it draws. By extension, the attention-enhancing effects of motivational salience are acquired through unsigned deviations from predicted value (Pearce and Hall, 1980; Fouragnan et al., 2017). We define such unsigned deviations from objectively or subjectively predicted value as objective or subjective risk prediction errors, given the mathematical identity of salience with risk prediction errors (Preuschoff et al., 2008). By being based on absolute value and symmetric, they differ from purely probability-based, nonmotivational definitions of expectancy violation (Brod et al., 2022) and from belief-dependent, nonsymmetric forms of surprise (Zhang and Rosenberg, 2024).

Traditionally, prediction errors are defined as the difference between expected and actual objective outcomes. However, prediction errors can also be computed for expectations and experience of subjectively evaluated outcomes. Such subjective prediction errors may more closely capture individual differences and track trial-by-trial variation in internal states and value processes driving adaptive behavior. In this regard, the distinction between subjective and objective value prediction errors follows on from the distinction between subjective and objective value (Rigoli et al., 2016), probability (Hsu et al., 2009), and risk (Holper et al., 2014; Kim et al., 2025). By analogy with the process of converting sensation into perception and cognition (Mesulam, 1998), it is conceivable that also value-related parameters undergo a similar transformation from more objective to more subjective judgment-related representations. While subjective value prediction errors have been associated with momentary well-being beyond purely statistical reinforcement learning (Rutledge et al., 2014) and with insula activity (Arulpragasam et al., 2018), the distinction between subjective and objective risk prediction errors remains to be elucidated.

For parametric trial-wise analyses, objective risk prediction errors at the time of outcomes were defined as the absolute difference between the value of the outcome (appetitive = +1, aversive = −1, neutral or no outcome = 0) and the expected value of the cue. Similarly, at the time of the cue, objective risk prediction error was the absolute difference between the expected value of the cue and the averaged expected values of all cues (which was 0). Thus, the objective cue risk prediction error corresponded to the absolute value of the cue.

Subjective risk prediction error at the time of the outcome was determined as the trial-wise absolute difference between outcome and cue rating. At the time of the cue, subjective risk prediction error was the absolute difference between the trial-wise cue rating and the average of all cue ratings in that block, specific for every participant. In some analyses (see below, GLM3), we also included the risk prediction error elicited by the preceding outcome at the time of the current cue (note that removing this regressor resulted in statistically the same conclusions concerning the other regressors).

### Analysis of behavioral and pupil data

#### Rating: absolute subjective value of appetitive, aversive, and neutral liquids

We averaged participants’ absolute ratings of liquids delivered with certainty (*p* = 1) during the task. If the absolute values of appetitive and aversive liquids remained matched, they should not differ significantly. To compare them, we conducted a paired sample *t* test across all participants.

#### Pupil dilation

Pupil dilation responses to cues and outcomes were normalized within each participant and block. For the cue, they were defined as the difference between pupil dilation at the motor response and precue baseline (Murphy et al., 2014). Pupil size at response was defined as the average pupil dilation from 250 ms before to 250 ms after the response, while baseline pupil dilation corresponded to the average pupil size in the 500 ms before cue onset (de Gee et al., 2017). For the outcome, pupil dilation was determined from 250 ms before to 1.5 s after the unconditioned stimulus (US) onset.

For a first-pass assessment of objective risk prediction errors that tightly controls for the nature of outcomes, we performed pairwise comparisons at the time of the outcomes. Specifically, we used paired *t* tests to compare the pupil dilation responses to outcomes that elicited an objective risk prediction error (after *p* = 0.5 cues) with the responses to outcomes that did not (i.e., occurring after *p* = 1 or *p* = 0 cues). The *α* level for statistical significance was set to 0.05 (two-tailed).

Next, we analyzed the cue [conditioned stimulus (CS)]- and outcome (US)-related pupil responses as a function of subjective risk prediction errors, using linear mixed-effects models:

$$\mathrm{Standardized}\;\mathrm{pupil}\;\mathrm{dilation}\;\mathrm{at}\;\mathrm{cue}=(\beta_{0}+\;b_{0j})\;+\;(\beta_{1}\;+\;b_{1j})\;\times \;|\mathrm{current}\;\mathrm{CS}\;\mathrm{PE}|\;+\;(\beta_{2}\;+\;b_{2j})\;\times \;|\mathrm{preceding}\;\mathrm{US}\;\mathrm{PE}|\;+\;e_{j},$$

$$\mathrm{Standardized}\;\mathrm{pupil}\;\mathrm{dilation}\;\mathrm{at}\;\mathrm{outcome}\;=\;(\beta_{0}\;+\;b_{0j})\;+\;(\beta_{1}\;+\;b_{1j})\;\times \;|\mathrm{US}\;\mathrm{PE}|\;+\;e_{j}.$$

In these models, *β*_0_ represents the intercept; *β*_1_ and *β*_2_ are fixed effects; *b*_0*j*_, *b*_1*j*_, and *b*_2*j*_ are random effects associated with the *j*-th subject; and *e_j_* is the residual error.

### Analysis of neuroimaging data

To assess risk prediction errors nonparametrically and parametrically, we modeled the imaging data at the participant level using three separate general linear models (GLMs) with different levels of granularity. The participant-wise (first level) contrast images resulting from these analyses were then evaluated at the group (second) level (all in SPM12).

#### Participant level analysis (first level)

All three models included onset regressors at five distinct time points: cue onset, behavioral response to cue, outcome onset, behavioral response to outcome, and swallow. The first model (GLM1) provided a first-pass assessment of objective risk prediction errors. It adopted a nonparametric approach to allow contrasting motivationally relevant outcomes that elicited an objective risk prediction error (after *p* = 0.5 cues) to the very same outcomes not eliciting an objective risk prediction error (after *p* = 0 or *p* = 1 cues). This analysis tightly controlled for the nature of outcomes and assessed objective risk prediction errors in four different cases, two in the aversive domain and two in the appetitive domain. We analyzed each of the four contrasts separately and then searched for overlap (see below).

The second model (GLM2) was parametric and applied two *z*-scored modulators to the outcome regressor, (1) objective risk prediction error and (2) subjective risk prediction error, both defined as described above (see above, Definition of objective and subjective risk/salience prediction errors). We interrogated the contrast images from the two types of prediction errors separately and contrasted them directly. Moreover, the CS onset regressor was parametrically modulated by subjective risk prediction errors.

The third model (GLM3) served to evaluate whether insula activity represented subjective risk prediction errors in a domain-general or domain-specific fashion. It was again parametric but modeled subjective risk prediction errors separately for the different domains (appetitive, aversive, neutral, and *p* = 0), both at the time of the outcome and the cue. Specifically, we used separate onset regressors for cues from each domain, modulated by their respective *z*-scored subjective risk prediction error induced by the current cue and the *z*-scored subjective risk prediction error induced by the preceding outcome. A model with only mean centering rather than full *z*-scoring yielded the same conclusions. We directly compared the processing of subjective risk prediction errors between cue and outcome time points, as well as between those elicited by the preceding outcome versus the current cue at the time of the cue. To allow all regressors to explain unique variance, orthogonalization of parametric regressors was turned off (Mumford et al., 2015) in both GLM2 and GLM3.

#### Group analysis (second level)

At the group level, we first used GLM1 to interrogate insula activity reflecting outcome-controlled objective risk prediction errors at the time of the outcome. To achieve this, we employed flexible factorial designs incorporating contrast images from the participant level. The analysis focused on the following contrasts to capture risk prediction errors regardless of whether outcomes were better or worse than predicted:

1. Better than predicted:
  • For appetitive liquid, we compared pleasant liquid delivered after *p* = 0.5 to the same liquid delivered after *p* = 1 cues.
  • In the aversive domain, we contrasted no liquid occurring after *p* = 0.5 against no liquid occurring after *p* = 0 cues.
2. Worse than predicted:
  • In the appetitive domain, we compared no liquid after *p* = 0.5 cues to no liquid after *p* = 0 cues.
  • For aversive liquid, we contrasted unpleasant liquid delivered after *p* = 0.5 cues to the same liquid delivered after *p* = 1 cues.

For GLM2 and GLM3, we examined the contrast images resulting from the parametric modulators. We also included regressors for day and participant to account for between-day and between-participant variability. We report our findings at the whole-volume level with a threshold of *p* < 0.05, corrected for familywise error (FWE) at the cluster level and a cluster-inducing voxel-level threshold of *p*_uncorr. _< 0.001. These criteria were applied to all voxels within the scanned volume. We used an insula mask from the Automated Anatomical Labeling Atlas 3 (AAL3; Rolls et al., 2020) to illustrate activity in regions that showed significant results after whole-volume correction.

## Results

### Absolute subjective value of liquids and pupil data

#### Matched absolute subjective value of appetitive and aversive liquids

In the calibration session, we selected appetitive and aversive liquids with matched absolute subjective values. To assess the relation between subjective ratings and willingness to pay (bids), we performed correlation analyses for each domain. We found that absolute ratings and absolute bids were significantly and similarly correlated in the appetitive (*r* = 0.58, *p* < 0.001) and aversive (*r* = 0.54, *p* < 0.001) domains (Fig. 1*c*). Within the main task, the average absolute ratings of deterministically delivered liquids showed no significant difference between aversive and appetitive liquids (*t*_(40)_ = 0.76, *p* = 0.45; Fig. 1*d*). Thus, the absolute value of appetitive and aversive liquids was matched also in the main task (and was higher than that of neutral liquids: Fig. 1*d*).

#### Pupil dilations reflect risk prediction errors

We first considered pupil responses to objective risk prediction errors in a categorical fashion. Specifically, we compared responses to the same kind of outcome when it was objectively surprising because it occurred after the *p* = 0.5 cue to when it was fully predicted because it occurred after the *p* = 1 or the *p* = 0 cue. We found that pupil dilation was significantly greater for unexpected outcomes (after *p* = 0.5 cues) compared with when the same outcome was fully expected (after *p* = 0 or *p* = 1 cues). This relation held when we compared surprising against fully predicted outcomes in the appetitive domain only (*t*_(40)_ = 3.06, *p* = 0.004 for reward delivered after *p* = 0.5 vs *p* = 1; *t*_(40)_ = 3.42, *p* = 0.002 for reward not delivered after *p* = 0.5 vs *p* = 0). The relation held similarly in the aversive domain (*t*_(40)_ = 2.28, *p* = 0.028 for punishment delivered after *p* = 0.5 vs *p* = 1; *t*_(40)_ = 2.45, *p* = 0.019 for punishment not delivered after *p* = 0.5 vs *p* = 0; Fig. 1*e*). Thus, pupil diameter increased in response to objective risk prediction errors (and did so to a similar degree), regardless of whether they occurred in the aversive or the appetitive domain.

Next, we considered pupil responses in a parametric fashion, as a function of subjective risk prediction errors at the time of the outcome (which was visually indicated only by a change in the color of the fixation cross). We determined subjective risk prediction errors as the absolute difference between outcome and cue rating in a trial- and participant-specific fashion. A linear mixed-effects regression model revealed a significant increase in pupil diameter with subjective risk prediction errors in the aversive (*β* = 0.04, *t*_(3543)_ = 2.71, *p* = 0.007; Fig. 1*f*) but not in the appetitive (*β* = 0.02, *t*_(3546)_ = 1.32, *p* = 0.19) domain (Fig. 1*f*). To analyze subjective risk prediction errors also at the time of the cue, we used the absolute difference between the cue rating and the average rating of all cues within the current block of trials for a given participant. We found a significant increase in pupil diameter with subjective risk prediction errors in both appetitive (*β* = 0.01, *t*_(3288)_ = 3.31, *p* = 9.5 × 10^−04^) and aversive (*β* = 0.18, *t*_(3269)_ = 9.40, *p* = 1.0 × 10^−20^ for aversive) domains (Fig. 1*f*). Thus, pupil dilation was related to both objectively and subjectively defined risk prediction errors.

### Neural results

#### Objective risk prediction error signals in the insula

**Figure 2.**
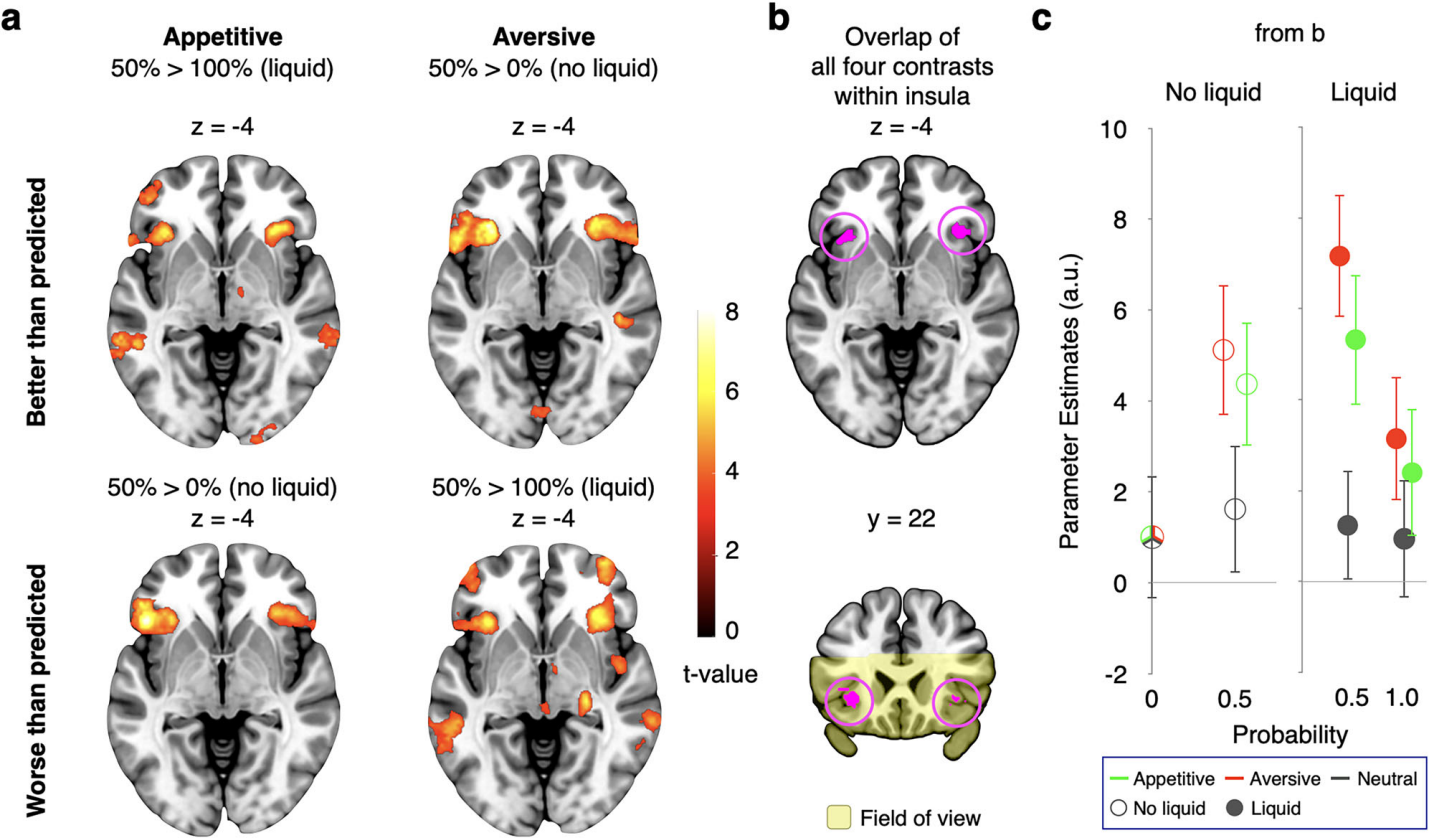
Relation between anterior insula activity and objective risk prediction errors at time of outcome (GLM1: nonparametric). ***a***, Anterior insula activity encoded basic forms of objective risk prediction errors, both when the outcomes were delivered and when they were withheld. Contrasts compared the same outcome when it elicited an objective risk prediction error (i.e., when it occurred after the *p* = 0.5 cue) to when it did not (i.e., when it occurred after the *p* = 1 or *p* = 0 cues). Accordingly, both in the appetitive (left) and aversive (right) domains, the anterior insula showed increased activity, regardless of whether outcomes were better than predicted (top) or worse than predicted (bottom), in line with processing risk as unsigned deviation from expectation. All four contrasts were separately whole-brain FWE cluster corrected (*p* < 0.05, with a cluster-inducing threshold of *p*_uncorr. _< 0.001). ***b***, Overlap (inclusive mask) of the four contrasts shown in ***a***, within the AAL3 insula mask. The field of view of the present study is indicated in light yellow in the coronal section at the bottom. ***c***, Illustration of neural activity (reflected by parameter estimates) within the cluster shown in ***b***, separately for no liquid (left) and for liquid conditions (right). Surprising neutral outcomes elicited weaker activation than surprising valenced outcomes. Data were extracted using GLM1, but no further statistical tests were run. Error bars indicate ±1 standard error of the mean.

First, we assessed objective risk prediction errors at the time of outcomes, using a categorical, nonparametric, approach (GLM1). To do so, we again formed contrasts for the same outcomes occurring with different levels of objective risk prediction error. The delivery of appetitive liquid in the *p* = 0.5 condition elicited a stronger anterior insula response than the delivery of the same appetitive liquid in the *p* = 1 condition (Fig. 2*a*, top left; see Table 1 for all regions). Moreover, the absence of appetitive liquid after the *p* = 0.5 cue activated the insula more strongly than the absence of liquid after the *p* = 0 cue (Fig. 2*a*, bottom left). Similarly, the anterior insula showed a stronger response to delivery and nondelivery of aversive liquid in the *p* = 0.5 condition than to the corresponding outcomes in the *p* = 1 and *p* = 0 conditions (Fig. 2*a*, top and bottom right). [As a side note, more posterior insula regions showed preferential encoding of motivationally relevant outcomes that were worse than predicted when compared with such outcomes that were better than predicted (data not shown).] Each of these four objective risk prediction error contrasts survived whole-volume familywise error (FWE) correction at the cluster level (cluster-inducing voxel-level threshold of *p*_uncorr. _< 0.001) and commonly activated the anterior insula (Fig. 2*b*). Plotting of the data within the common insula voxels for illustration revealed similar activation levels in the appetitive and aversive domains but little activation for surprising neutral outcomes (Fig. 2*c*). This pattern of activity is compatible with objective risk prediction error processing.

**Table 1.** Brain regions encoding components of objective risk prediction errors at the time of outcomes (GLM1: nonparametric)

Contrast	Region	Cluster size (# voxels)	Mean *t* statistic	Peak MNI coordinates		
				*x*	*y*	*Z*
Appetitive 50% > appetitive 100% (liquid)	Frontal_Inf_Tri_L, extending into **Insula_L (ant)**	1,350	8.14	−36	38	8
			6.09	−56	18	2
			5.80	−54	20	22
	Caudate_L	121	6.71	−8	8	4
	**Insula_R (ant)**	231	6.69	30	24	−4
	Temporal_Mid_R	178	6.60	70	−36	−10
	Left cerebral white matter^a^	385	6.19	−50	−38	−12
	Temporal_Mid_L		4.33	−56	−52	2
	Temporal_Inf_L		4.00	−56	−56	−22
	Thal_MDl_R	155	5.30	8	−16	6
	Cerebellum_Crus1_R	80	5.00	12	−84	−24
	Cerebellum_Crus1_L	106	3.92	−32	−72	−28
	Occipital_Mid_R	84	4.56	34	−82	0
	Calcarine_R		4.47	22	−98	−2
	Cerebellum_Crus2_L	73	4.37	−12	−84	−30
Aversive 50% > 0% (no liquid)	Cuneus_R	1,491	11.12	14	−96	12
	Lingual_L		7.09	0	−82	4
	Occipital_Mid_L		5.22	−12	−102	6
	Frontal_Inf_Orb_2_L extending into **Insula_L (ant)**	1,309	8.93	−34	22	−10
	Frontal_Inf_Tri_L		8.92	−52	22	2
	Frontal_Inf_Oper_L		4.13	−60	8	16
	Frontal_Inf_Orb_2_R	1,038	7.41	48	22	−10
	**Insula_R (ant)**		6.63	28	18	−14
	Right cerebral white matter^a^		4.04	26	20	8
	Cerebellum_8_L	73	6.25	−34	−36	−42
	ACC_pre_L	400	5.87	−6	38	14
	ACC_pre_R		4.69	14	40	16
	Temporal_Sup_R	112	5.69	46	−28	−2
	ACC_sup_L	62	5.46	0	18	22
	SN_pc_L	61	5.13	−6	−12	−12
	Brainstem^a^ extending into LC	99	4.53	8	−36	−32
Appetitive 50% > 0% (no liquid)	Frontal_Inf_Orb_2_L	1,067	8.15	−46	24	−4
	**Insula_L (ant)**		6.40	−28	16	−14
	Cuneus_R	264	7.96	18	−100	8
	ACC_sup_L	402	7.42	−8	36	18
	ACC_pre_R		6.20	12	40	14
	ACC_pre_L		3.75	−6	48	2
	Right cerebral white matter^a^	570	6.75	32	32	0
	**Insula_R (ant)**		5.05	34	16	−18
	Frontal_Inf_Tri_R		4.33	52	22	−2
Aversive 50% > aversive 100% (liquid)	Temporal_Mid_L	452	6.92	−58	−42	−10
	Frontal_Inf_Tri_L extending into **Insula_L (ant)**	1,438	6.87	−34	26	−2
			5.40	−48	30	18
	Frontal_Mid_2_L		4.99	−44	56	−2
	Right cerebral white matter^a^	1,407	6.61	24	16	−14
	Frontal_Mid_2_R extending into **Insula_R (ant)**		6.26	36	56	−2
	Right cerebral white matter^a^		5.96	36	32	0
	Temporal_Inf_R	402	6.61	62	−40	−16
	Thal_MDm_R	501	6.36	6	−12	2
	Caudate_R		4.55	14	6	14
	Right cerebral white matter^a^	152	6.20	26	−24	−4
	Precuneus_L	207	5.40	−12	−52	24
	Cingulate_Post_R		4.65	8	−40	14
	ACC_sup_L	70	5.18	−6	22	22
	Cerebellum_Crus1_L	58	5.06	−10	−78	−26
	SN_pc_R	114	4.97	0	−14	−12
	**Insula_R (mid)**	82	4.80	46	−4	−4
	Brainstem^a^	76	4.45	6	−28	−16

All activations are whole-brain familywise error rate (FWE) corrected at the cluster level, *p* < 0.05; cluster-inducing voxel threshold *p*_uncorr. _< 0.001. Apart from ^a^ (see below), gray matter labels are from Automated Anatomical Labelling Atlas 3 (AAL3; Rolls et al., 2020).

Abbreviations: ant, anterior; mid, middle.

a Label from Harvard-Oxford Atlas (region not defined in the AAL atlas).

**Figure 3.**
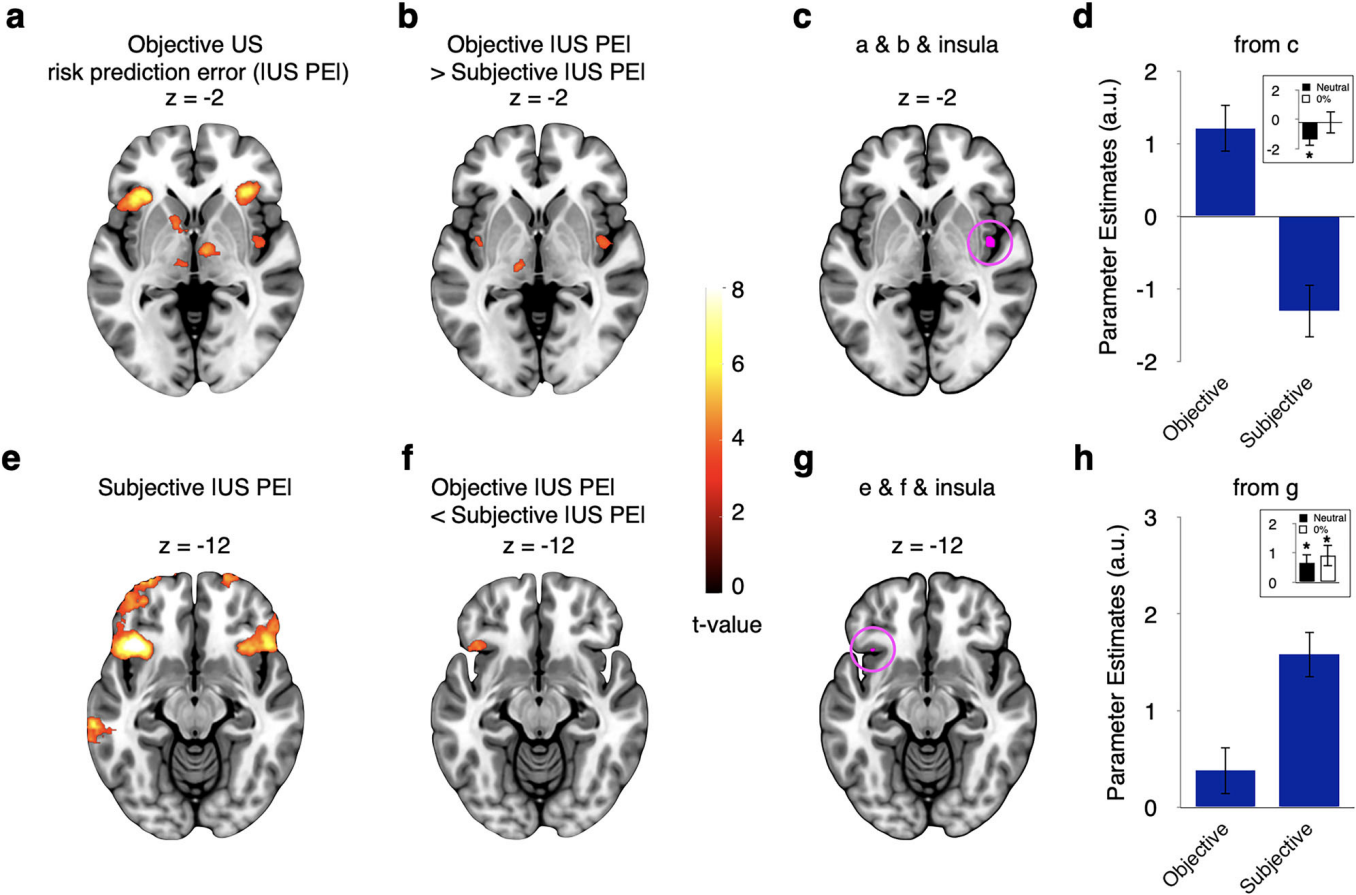
Relation between insula activity and objective or subjective risk prediction errors at the time of outcome (GLM2: parametric, domain-combined). ***a***, Regions showing a positive relation with objective parametric risk prediction errors at the time of the outcome (|US PE|). ***b***, Direct contrasts revealed a stronger relation to objective than subjective risk prediction errors in the mid-insula. ***c***, Activity shown in ***b*** inclusively masked with activity shown in ***a***, within AAL3 insula mask. ***d***, Illustration of association between mid-insula activity and objective and subjective risk prediction errors within the cluster shown in ***c***. Inset, The cluster in ***c*** shows no positive relation to subjective risk prediction errors in neutral and 0% conditions (from GLM3). ***e***, Regions showing a positive relation to trial-wise subjective parametric risk prediction errors at the time of the outcome. ***f***, Direct contrasts revealed a stronger relation to subjective than objective risk prediction errors in the anterior insula. ***g***, Activity shown in ***f*** inclusively masked with activity shown in ***e***, within the AAL3 insula mask. ***h***, Visualization of association between anterior insula activity and objective and subjective risk prediction errors within cluster shown in ***g***. Inset, In contrast to the cluster in ***c***, the cluster in ***g*** shows a positive relation to subjective risk prediction errors in both neutral and 0% conditions (from GLM3). Analyses shown in ***a***, ***b***, ***e***, and ***f*** were whole-brain FWE cluster corrected. For ***d*** and ***h***, data were extracted using GLM2, but no further statistical tests were run. Error bars represent ±1 standard error of the mean. Abbreviations: |US PE|, absolute unconditioned stimulus prediction error, i.e., risk prediction error at outcome.

Next, we asked whether a parametric assessment of an objective risk prediction error signal would replicate the findings of the nonparametric analysis. Therefore, GLM2 parametrically modulated the outcome onset regressor by the objective unsigned value difference between outcome and prediction in each trial. This modulator identified the same anterior insula region as the pairwise contrasts of GLM1 (Fig. 3*a*, whole-volume FWE corrected at the cluster level; see Table 2 for all regions). In addition, it also identified a region in the mid-insula, where activity increased with the size of the objective risk prediction error. Thus, the parametric approach converged with, and extended, the nonparametric approach.

**Table 2.** Brain regions encoding objective and subjective risk prediction error signals at outcomes, and subjective risk prediction error signals at cues (GLM2: parametric))

Contrast	Region	Cluster size (# voxels)	Mean *t* statistic	Peak MNI coordinates		
				*x*	*y*	*z*
Objective |US PE|	Frontal_Inf_Orb_2_R extending into **Insula_R (ant)**	513	7.58	40	26	−8
	Right cerebral white matter^a^		3.50	30	36	10
	Right cerebral white matter^a^	492	6.97	6	−12	−10
	Right cerebral white matter^a^ extending into SN_pc_R		4.83	14	2	10
	Thal_IL_L		4.82	−12	−18	2
	**Insula_L (ant)**	422	6.93	−36	22	−2
	Cerebellum_Crus1_L	258	5.93	−36	−68	−34
	Frontal_Inf_Oper_R	172	4.64	54	14	6
	**Insula_R (mid)**		4.62	40	−8	2
Objective |US PE| > subjective |US PE|	Temporal_Sup_R	966	5.55	64	−12	10
	**Insula_R (mid)**		4.91	42	−10	6
	Temporal_Sup_R		4.82	60	−32	12
	Cerebellum_Crus1_L	124	5.28	−30	−58	−38
	Cerebellum_6_R	72	5.13	22	−66	−22
	**Insula_L (mid)**	84	5.08	−36	−6	2
	Thal_IL_L	66	5.05	−12	−20	0
Subjective |US PE|	Frontal_Inf_Orb_2_L extending into **Insula_L (ant)**	2,210	11.14	−38	24	−10
	Frontal_Inf_Tri_L		7.23	−52	30	4
	Frontal_Mid_2_L		6.30	−38	50	−8
	ACC_sup_L	2,340	7.69	2	20	22
	ACC_pre_L		6.95	−4	40	24
	Frontal_Sup_Medial_R		5.84	4	58	16
	Temporal_Mid_L	345	7.60	−64	−28	−10
	Left cerebral white matter^a^		3.51	−48	−40	−2
	Frontal_Inf_Orb_2_R extending into **Insula_R (ant)**	1,339	7.01	48	26	−10
	OFCpost_R		6.01	28	22	−22
	Frontal_Inf_Tri_R		5.54	62	22	16
	Temporal_Inf_L	245	6.36	−52	6	−38
			4.32	−56	−12	−30
	Temporal_Mid_R	351	6.07	48	−30	−6
			4.51	62	−42	2
	Occipital_Sup_R	168	5.36	22	−98	14
	Occipital_Mid_R		3.95	40	−86	8
	Temporal_Mid_L	67	5.04	−48	−46	4
Objective |US PE| < subjective |US PE|	ACC_pre_L	739	5.19	−10	38	22
	Frontal_Sup_Medial_R		5.07	2	60	6
	Frontal_Sup_2_L		4.57	−20	64	0
	OFCpost_L extending into **Insula_L (ant)**	111	5.06	−40	20	−14
	Frontal_Inf_Tri_R	144	4.89	56	36	4
	Occipital_Mid_R	68	4.75	36	−84	8
Subjective |CS PE|	Lingual_L	1,854	15.72	−10	−84	−8
	Cuneus_R		13.10	14	−90	16
	Occipital_Sup_L		5.14	−10	−102	8
	Temporal_Mid_L	1,373	10.64	−60	−42	2
			5.55	−64	−24	−10
			5.22	−56	−66	14
	OFCpost_L	1,411	8.47	−40	30	−18
	Frontal_Inf_Tri_L		7.66	−56	28	4
	Temporal_Pole_Sup_L extending into **Insula_L (ant)**		5.99	−48	12	−22
	**Insula_R (ant)**	727	7.22	30	16	−18
	OFCant_R		6.54	40	52	−14
	Frontal_Inf_Tri_R		5.41	50	28	2
	Fusiform_R	84	7.15	32	−78	−10
	Rectus_L	797	6.61	−4	52	−18
	Frontal_Med_Orb_R		6.01	8	60	−4
	Frontal_Sup_2_L		5.33	−22	60	−10
	Temporal_Sup_R	398	6.14	52	−26	−4
	Temporal_Mid_R		5.51	68	−38	−8
	Frontal_Sup_Medial_L	385	6.04	−12	46	18
	ACC_pre_R		4.27	8	46	10
	Temporal_Inf_L	150	5.72	−46	2	−40
Subjective |US PE| > subjective |CS PE|	Lingual_L	324	6.99	−4	−70	2
	Frontal_Inf_Orb_2_L extending into **Insula_L (ant)**	253	6.52	−36	22	−10
	ACC_pre_L	225	6.04	−10	38	22
	ACC_pre_R		4.28	10	46	22
	Putamen_R	112	5.84	20	6	−8
	Lingual_R	91	4.65	16	−76	−2
	Olfactory_L	65	4.47	−14	10	−14
Subjective |US PE| < subjective |CS PE|	Calcarine_L	436	6.87	−2	−84	0
	Cuneus_R		5.89	10	−90	16
	Brainstem^a^	69	4.66	10	−20	−26
	ParaHippocampal_L	66	4.48	−26	−18	−24

Activations were whole-brain familywise error (FWE) cluster-level corrected, *p* < 0.05; cluster-inducing voxel threshold *p*_uncorr. _< 0.001. Apart from ^a^ (see below), gray matter labels are from Automated Anatomical Labelling Atlas 3 (AAL3; Rolls et al., 2020).

Abbreviations: |US PE|, absolute unconditioned stimulus prediction error, i.e., risk prediction errors elicited by outcomes; |CS PE|, absolute conditioned stimulus prediction error, i.e., risk prediction error elicited by cues; ant, anterior; mid, middle.

a Label from Harvard-Oxford Atlas (region not defined in the AAL atlas).

#### Dissociation of subjective and objective risk prediction error signals in the insula

To investigate subjective risk prediction error processing, GLM2 parametrically modulated the outcome onset regressor also with the absolute difference between the outcome rating and the cue rating in each trial. Objective risk prediction errors were another parametric modulator of the outcome regressor and they correlated with subjective risk prediction errors (*r* = 0.58, *p* = 0.004). By extension, a potential concern in the analysis of the subjective risk prediction error is multicollinearity, which can complicate the interpretation of regression results due to the correlation with objective risk prediction errors. To assess this, we computed the variance inflation factors (VIFs) for each block to determine the extent of collinearity, using a conservative threshold of VIF > 5 as indicative of significant multicollinearity (O’Brien, 2007). We found that the mean VIF was 1.74 (SEM = 0.10), indicating that multicollinearity is unlikely to be a concern for this analysis.

The two insula regions identified by the objective parametric modulator showed a different relation to the subjective parametric modulator. Direct comparison revealed that activity in the mid-insula was more strongly related to objective than subjective risk prediction errors (Fig. 3*b*–*d*; whole-volume FWE corrected at the cluster level; see Table 2 for all regions). In contrast, the subjective risk prediction error modulator identified the anterior insula (Fig. 3*e*; whole-volume FWE corrected at the cluster level; see Table 2 for all regions), i.e., the same region as that identified by the objective risk prediction error modulator, with activity extending also anteriorly into the frontal cortex. This and subsequent results remained similar and statistically significant when we additionally included a parametric modulator modeling signed value prediction error. Importantly, direct comparison showed stronger encoding of subjective than objective risk prediction error by the left anterior insula (Fig. 3*f*–*h*). These findings indicate a double dissociation of subjective and objective risk prediction error coding along the anterior–posterior axis of the insula.

A primary difference between objective and subjective risk prediction error (which also limits the collinearity of the two modulators) is that subjective but not objective risk prediction errors occur also after cues predicting neutral liquid and even after cues predicting no outcome. We therefore interrogated these conditions separately (GLM3) and found that insula activity indeed correlated positively with subjective risk prediction errors in the anterior but not in the mid-insula cluster (insets of Fig. 3*d*,*h*). Thus, core regions of the salience network (anterior insula, anterior cingulate cortex: Table 2) significantly and preferentially encode subjective rather than objective risk prediction errors at the time of outcomes. Based on these findings, and because the anterior rather than the mid-insula constitutes the hub of the salience network, we focused on subjective risk prediction error encoding by the anterior insula in all subsequent analyses.

#### Subjective risk prediction error signal also at time of cues in the anterior insula

**Figure 4.**
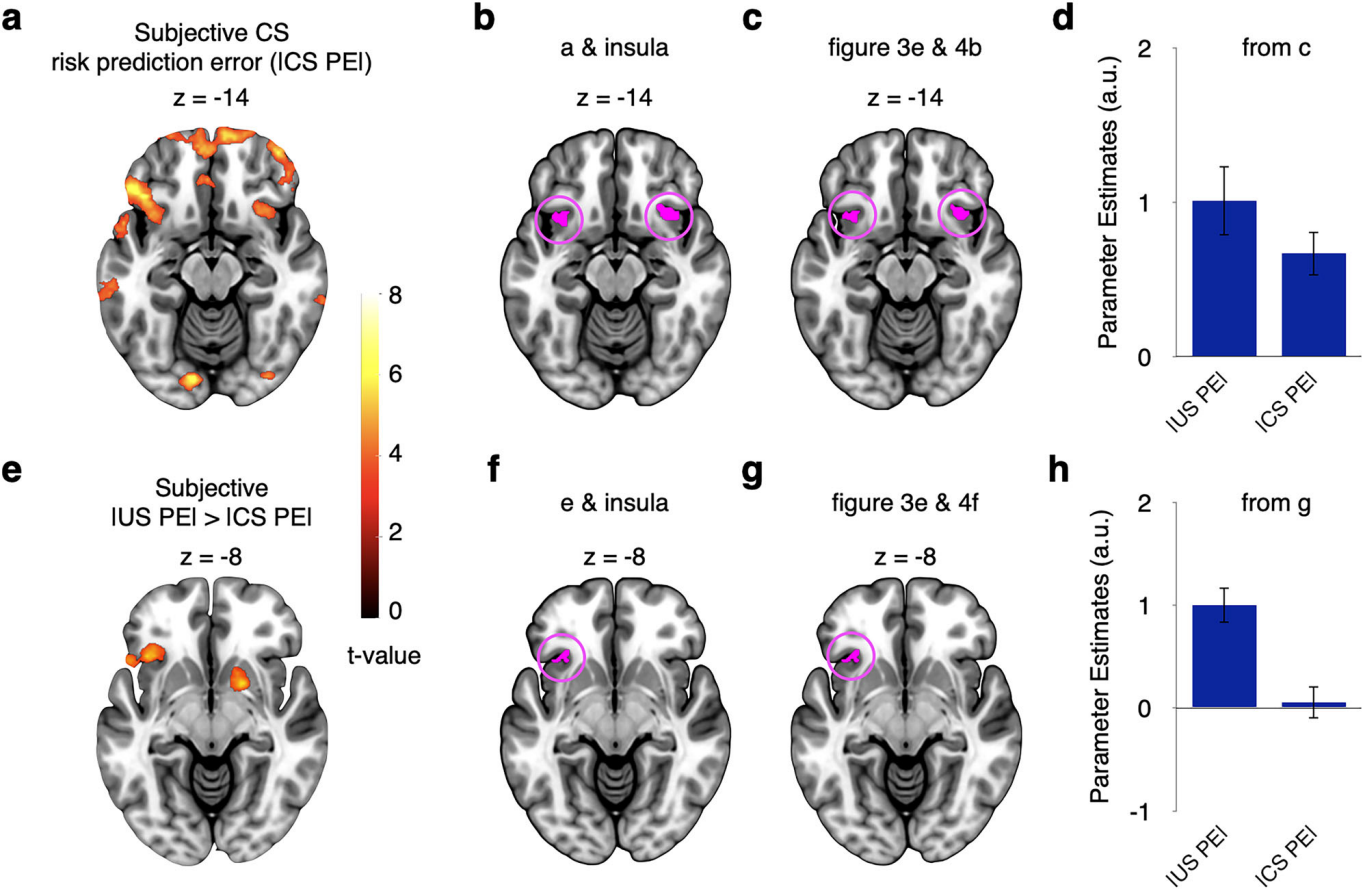
Subjective risk prediction error responses at the time of cue and comparison with time of outcome (GLM2: parametric, domain-combined). ***a***, Activations related to trial-wise subjective risk prediction errors at the time of cue (CS). Subjective risk prediction errors induced by cues were determined as the absolute difference between the cue rating in a given trial and the participant- and block-specific average rating of all cues. ***b***, The regions encoding subjective cue risk prediction errors identified in ***a*** were partly located within the AAL3 insula mask. ***c***, Anterior insula regions encoding a parametric subjective risk prediction error both at the time of the outcome (from Fig. 3*e*) and the cue (from ***a***). ***d***, Illustration of activity from cluster shown in ***c***. ***e***, Regions showing stronger encoding of subjective risk prediction errors at the time of the outcome than at the time of the cue. ***f***, Activity of ***e***, limited to AAL3 insula mask. ***g***, The cluster in ***e*** also overlapped with regions showing a positive relation to subjective risk prediction errors at the time of the outcome (from Fig. 3*e*), suggesting that the finding is not primarily driven by a negative relation to cue risk prediction error. ***h***, This conclusion is corroborated by visualization of activity in the insular cluster from ***g***. Analyses shown in ***a*** and ***e*** were whole-brain FWE cluster corrected. For ***d*** and ***h***, data were extracted using GLM2, but no further statistical tests were run. Error bars represent ±1 standard error of the mean. Abbreviations: |CS PE|, absolute conditioned stimulus prediction error, i.e., risk prediction error induced by cues; |US PE|, absolute unconditioned stimulus prediction error, i.e., risk prediction error induced by outcomes.

Next, we interrogated the generality of subjective risk prediction error coding in the anterior insula. Specifically, we asked whether the encoding of subjective risk prediction errors in the anterior insula occurs also at the time of the cue, rather than only at the time of the outcome. The question is also relevant because trial-based learning theories expect encoding of prediction errors only at the outcome, whereas a real-time learning model expects it also at the time of the cue. GLM2 therefore parametrically modulated the cue onset regressor with the absolute difference between the cue rating in each trial and the participant-specific and block-specific average rating of all cues. Interrogating this regressor identified clusters bilaterally including the anterior insula and adjacent frontal cortex (Fig. 4*a*,*b*; whole-volume FWE corrected at the cluster level; see Table 2 for all regions), as well as the anterior cingulate cortex.

To assess whether indeed the same regions encoded risk prediction errors at the time of the cue and the time of the outcome, we used inclusive masking. This analysis revealed common relations to subjective risk prediction errors in the ventral anterior insula at both time points (Fig. 4*c*,*d*). A direct comparison also revealed significantly stronger encoding of subjective risk prediction errors at the time of the outcomes than at the time of the cues in a more dorsal part of the anterior insula (Fig. 4*e*,*f*). As expected, this cluster also overlapped (Fig. 4*g*) with the anterior insula region encoding subjective risk prediction errors at the time of the outcome identified above (Fig. 3*e*). Thus, some anterior insula regions preferentially encode risk prediction errors at the time of the outcomes (Fig. 4*h*) rather than the cues. In principle, this finding is compatible with higher motivational relevance of outcomes than of cues predicting these outcomes. However, the domain-specific analysis to which we turned next provided a different interpretation.

#### Subjective risk prediction error signals in the anterior insula depend on event timing

**Figure 5.**
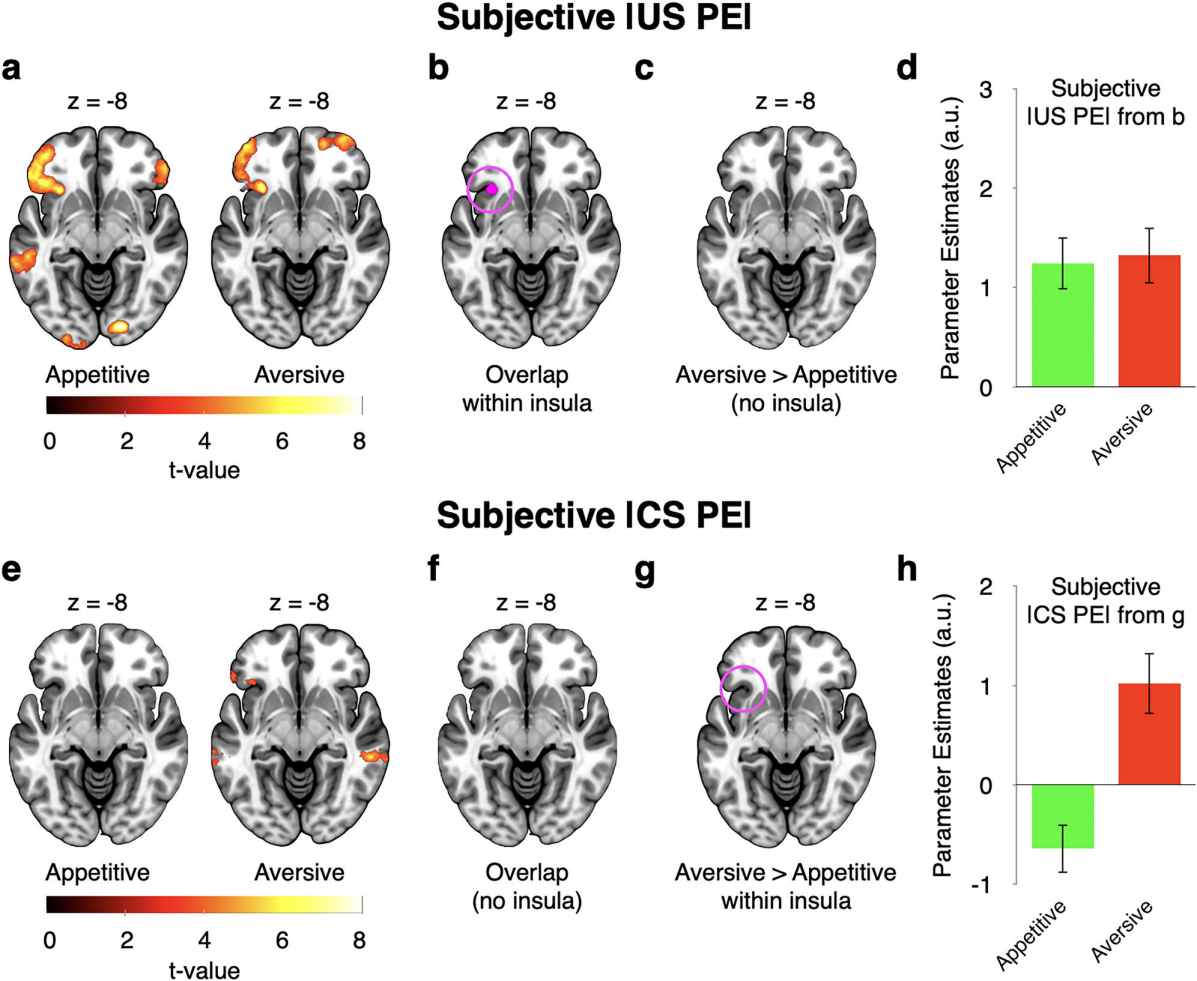
Domain generality and specificity of subjective risk prediction error responses (GLM3: parametric, domain-separated). ***a***, Regions encoding parametric subjective risk prediction errors at the time of outcomes in the appetitive (left) and aversive (right) domains. ***b***, Overlap of the appetitive and aversive domains from ***a***, within the AAL3 insula mask. ***c***, No significant domain difference in the encoding of subjective risk prediction errors at the time of outcomes within the insula (only an occipital region showed a difference at our whole-brain corrected threshold). ***d***, Illustration of activity from cluster shown in ***b***. ***e***, Regions encoding parametric subjective risk prediction errors at the time of cues. The anterior insula encoded cue-induced risk prediction errors only in the aversive, but not in the appetitive, domain. ***f***, Accordingly, the two contrasts from ***e*** did not result in any overlap in the insula. ***g***, Direct comparison of aversive versus appetitive subjective risk prediction errors at the time of cues revealed a significant difference in the anterior insula. Note that the peak of the cluster was more dorsal, but for consistency with the remainder of the figure, we display the insula section at the same ventral level. ***h***, Illustration of activity from the cluster in ***g***. Analyses shown in ***a*** and ***e*** were whole-brain FWE cluster corrected. For ***d*** and ***h***, data were extracted using GLM3, but no further statistical tests were run. Error bars represent ±1 standard error of the mean.

To interrogate the nature of subjective risk prediction error signals further, we modeled them (GLM3) separately in the appetitive and the aversive domains, both at the time of outcomes and at the time of cues. Converging with the solid encoding of subjective risk prediction errors at the time of the outcomes, anterior insula (and adjacent frontal cortex) activity correlated with subjective risk prediction errors induced by outcomes in the appetitive and the aversive domains (Fig. 5*a*; whole-volume FWE corrected at the cluster level; see Table 3 for all regions). The subjective risk prediction error signals in the two domains overlapped within the anterior insula (Fig. 5*b*). Moreover, there was no significant difference in subjective risk prediction error processing between the appetitive and the aversive domains (Fig. 5*c*,*d*). Thus, at the time of outcomes, the anterior insula appears to encode subjective risk prediction errors in a domain-general fashion.

**Table 3.** Brain regions encoding subjective risk prediction errors by domain at the time of outcomes or cues (GLM3: parametric)

Contrast	Region	Cluster size (# voxels)	Mean *t* statistic	Peak MNI coordinates		
				*x*	*y*	*z*
Subjective |US PE| (appetitive)	Lingual_R	295	9.52	18	−88	−6
	**Insula_L (ant)**	1,963	7.76	−30	20	−8
	Frontal_Inf_Tri_L		7.68	−54	22	10
	Frontal_Mid_2_L		7.47	−40	48	−8
	Temporal_Mid_L	430	6.84	−54	−34	−4
	Frontal_Inf_Orb_2_R	331	6.29	54	38	−6
	Occipital_Mid_L	207	5.80	−26	−100	−4
Subjective |US PE| (aversive)	**Insula_L (ant)**	1,270	8.33	−30	22	−6
	Frontal_Inf_Tri_L		6.49	−58	22	8
			6.41	−44	42	−2
	Frontal_Sup_2_R	1,089	6.05	34	60	−8
	Frontal_Sup_2_L		5.95	−18	62	20
	Frontal_Sup_Medial_R		4.97	16	60	10
	ACC_pre_R	66	5.32	4	44	24
	Temporal_Inf_L	107	5.07	−52	−4	−42
	Temporal_Pole_Mid_R	116	4.74	54	14	−30
	OFCpost_R		4.45	34	24	−26
	Calcarine_L	68	4.72	−30	22	−6
Subjective |CS PE| (appetitive)	n.s.					
						
Subjective |CS PE| (aversive)	**Insula_L (ant)**	1,270	8.33	−30	22	−6
	Frontal_Inf_Tri_L		6.49	−58	22	8
			6.41	−44	42	−2
	Frontal_Sup_2_R	1,089	6.05	34	60	−8
	Frontal_Sup_2_L		5.95	−18	62	20
	Frontal_Sup_Medial_R		4.97	16	60	10
	ACC_pre_R	66	5.32	4	44	24
	Temporal_Inf_L	107	5.07	−52	−4	−42
	Temporal_Pole_Mid_R	116	4.74	54	14	−30
	OFCpost_R		4.45	34	24	−26
	Calcarine_L	68	4.72	−16	−102	−4
Subjective |US PE| > |CS PE| (appetitive)	Frontal_Inf_Tri_L	816	6.44	−52	22	10
	**Insula_L (ant)**		5.85	−28	20	−8
	Frontal_Inf_Orb_2_L		5.40	−44	40	−6
	Calcarine_L	279	6.28	−12	−104	−4
	Lingual_R	67	6.00	20	−88	−6
	Frontal_Sup_2_L	103	5.77	−24	56	12
	Temporal_Mid_L	296	5.41	−50	−34	−4
Subjective |US PE| > |CS PE| (aversive)	Calcarine_L	112	6.62	−14	−100	−4
	Right cerebral white matter^a^	64	4.59	20	52	−6
	Frontal_Sup_2_R		3.79	38	60	−12

Activations were whole-brain familywise error (FWE) cluster-level corrected, *p* < 0.05; cluster-inducing voxel threshold *p*_uncorr. _< 0.001. Apart from ^a^ (see below), gray matter labels are from Automated Anatomical Labelling Atlas 3 (AAL3; Rolls et al., 2020).

Abbreviations: |US PE|, absolute unconditioned stimulus prediction error, i.e., risk prediction errors elicited by outcomes; |CS PE|, absolute conditioned stimulus prediction error, i.e., risk prediction error elicited by cues; ant, anterior.

a Label from Harvard-Oxford Atlas (region not defined in the AAL atlas).

A different picture emerged at the time of cues. At this time point, the anterior insula processed subjective risk prediction errors only in the aversive domain, but not in the appetitive domain (Fig. 5*e*,*f*; whole-volume FWE corrected at the cluster level; see Table 3 for all regions). Moreover, it processed subjective risk prediction errors induced by cues significantly more strongly in the aversive than the appetitive domain (Fig. 5*g*,*h*). These findings suggest that at the time of cues, the anterior insula encodes subjective risk prediction errors preferentially in the aversive domain, i.e., in a domain-specific fashion.

**Figure 6.**
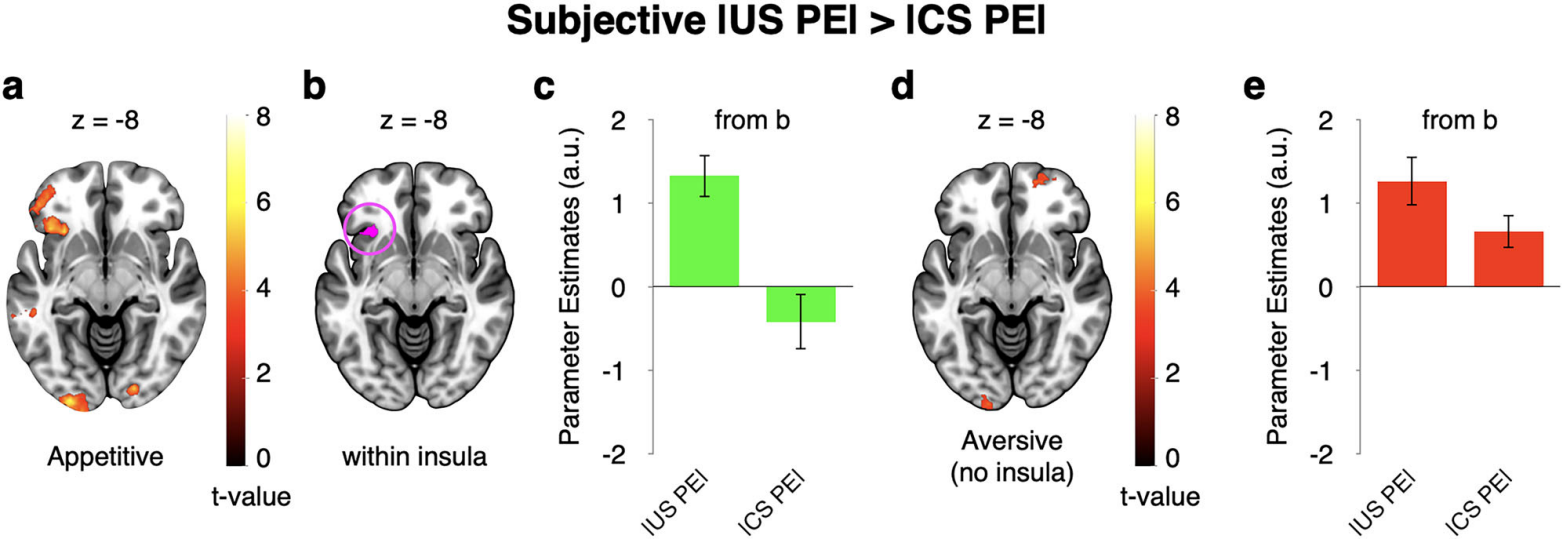
Subjective risk prediction error responses in the anterior insula are preferentially (commonly) encoded at the time of outcomes (cues) in the appetitive (aversive) domain (GLM3: parametric, domain-separated). ***a***, Regions showing stronger relation to subjective risk prediction error at the time of outcomes than at the time of cues in the appetitive domain. ***b***, Activity from ***a*** shown within AAL3 insula mask. ***c***, Illustration of activity from cluster shown in ***b***. ***d***, In contrast, in the aversive domain, there was no significant difference in subjective risk prediction error signals between the time of outcomes and the time of cues. ***e***, Illustration of activity within cluster shown in ***b***. The maps in ***a*** and ***d*** were whole-brain FWE cluster corrected. For ***c*** and ***e***, data were extracted using GLM3, but no further statistical tests were run. Error bars represent ±1 standard error of the mean.

To further assess temporal specificity, we next used GLM3 to directly compare subjective risk prediction error signals at the time of the outcome with those at the time of the cue, separately for the appetitive and aversive domains. In agreement with domain specificity, anterior insula activity encoded subjective risk prediction errors significantly more strongly at the time of outcomes than at the time of cues in the appetitive domain (Fig. 6*a*–*c*). In contrast, it processed them similarly and not significantly differently at the two time points in the aversive domain (Fig. 6*d*,*e*). Thus, the anterior insula appears to process subjective risk prediction errors differently in the appetitive and aversive domains.

#### Continued encoding of preceding outcome-induced risk prediction errors at time of current cue in the appetitive but not the aversive domain

**Figure 7.**
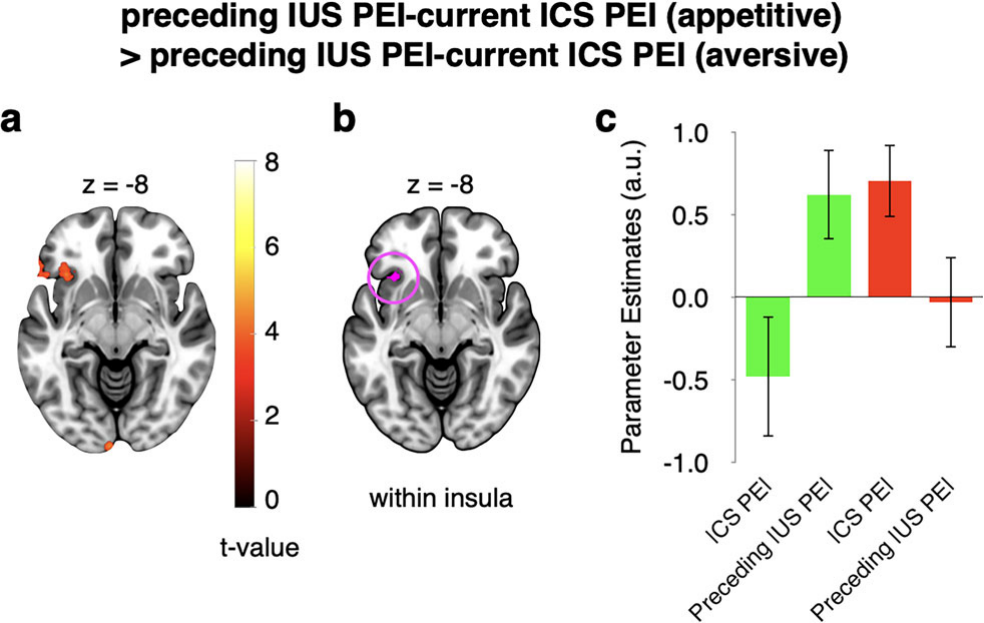
Continued encoding of preceding outcome-induced risk prediction errors at the time of current cue in the appetitive but not the aversive domain (GLM3: parametric, domain-separated). ***a***, Regions showing stronger relation to preceding |US PE| compared with current |CS PE| in appetitive than in the aversive domain at the time of the cues (interaction). ***b***, These regions located partly within the AAL3 insula mask. ***c***, Illustration of activity from cluster shown in ***b***. The maps in ***a*** were whole-brain FWE cluster corrected. For ***c***, data were extracted using GLM3, but no further statistical tests were run. Error bars represent ±1 standard error of the mean. Abbreviations: |CS PE|, absolute conditioned stimulus prediction error, i.e., risk prediction error induced by cues; |US PE|, absolute unconditioned stimulus prediction error, i.e., risk prediction error induced by outcomes.

We reasoned that a possible explanation for the preferential encoding of subjective risk prediction errors in the aversive domain could be that the anterior insula may update cue-related risk signals in the aversive domain more strongly than in the appetitive domain. Compatible with this notion, GLM3 showed that the parametric subjective risk prediction error elicited by preceding outcomes continued to be encoded at the time of the subsequent cue significantly more strongly in the appetitive than in the aversive domain (*p* < 0.05, whole-volume FWE corrected at the cluster level; Fig. 7; see Table 4 for all regions). Moreover, a direct comparison within the appetitive domain revealed a significantly stronger encoding of the subjective risk prediction error elicited by the preceding outcome than by the current cue (Table 4). Plotting the effects indicated that at the time of the cue, the anterior insula encoded primarily the subjective risk prediction error elicited by the current cue in the aversive domain and the subjective risk prediction error elicited by the preceding outcome in the appetitive domain (Fig. 7*c*). These findings converge with the notion of reduced updating of risk predictions in the appetitive domain.

**Table 4.** Brain regions with domain-dependent continued encoding of subjective risk prediction errors elicited by preceding outcomes rather than encoding of subjective risk prediction error elicited by current cues (GLM3: parametric)

Contrast	Region	Cluster size (# voxels)	Mean *t* statistic	Peak MNI coordinates		
				*x*	*y*	*z*
Subjective [preceding |US PE|–current |CS PE| (appetitive)] > [preceding |US PE|–current |CS PE| (aversive)]	Frontal_Inf_Tri_L	740	6.58	−44	22	18
	Frontal_Inf_Orb_2_L		5.71	−50	24	−12
	Temporal_Pole_Sup_L		4.41	−30	18	−30
	Temporal_Mid_L	481	6.26	−56	−48	4
			3.91	−58	−24	−4
	Frontal_Sup_2_L	115	5.75	−24	52	18
	Calcarine_R	127	5.75	14	−92	6
	Caudate_L	125	5.31	−10	8	8
	**Insula_L (ant)**	87	4.99	−38	−12	20
	ACC_sup_L	93	4.80	−4	32	24
Subjective [preceding |US PE|–current |CS PE| (appetitive)] < [preceding |US PE|–current |CS PE| (aversive)]	Lingual_R	66	5.88	10	−84	−2

Activations were whole-brain familywise error (FWE) cluster-level corrected, *p* < 0.05; cluster-inducing voxel threshold *p*_uncorr. _< 0.001. Gray matter labels are from Automated Anatomical Labelling Atlas 3 (AAL3; Rolls et al., 2020).

Abbreviations: |US PE|, absolute unconditioned stimulus prediction error, i.e., risk prediction errors elicited by outcomes; |CS PE|, absolute conditioned stimulus prediction error, i.e., risk prediction error elicited by cues; ant, anterior.

## Discussion

Our study reveals the nature of risk processed by the insula. While the anterior insula preferentially encoded errors in the prediction of subjectively risky events, the mid-insula specifically encoded objective risk prediction errors. Moreover, although the anterior insula processed subjective risk prediction errors both at the time of outcomes and at the time of cues, the responses were more domain-general at the time of outcomes than at the time of cues. At the time of the cues, the anterior insula encoded subjective risk prediction errors induced by the current cue preferentially in the aversive rather than the appetitive domain. In contrast, it encoded the prediction errors induced by the preceding outcomes preferentially in the appetitive rather than the aversive domain. These findings provide an explanation for the coexistence of domain generality and domain specificity within the anterior insula. More generally, they support the unifying framework that the anterior insula processes affect, risk, and interoception (Singer et al., 2009) and reveal how these functions are integrated. By directly comparing subjective to objective risk processing and demonstrating domain-specific updating, our study thus advances our understanding of the role of the insula.

### Functions of risk/salience prediction errors

Behaviorally, risk/salience prediction errors can be used to facilitate learning by increasing attention and energizing behavior to the stimuli they are associated with (Mackintosh, 1975; Pearce and Hall, 1980; Tremblay and Schultz, 1999; Mendelsohn et al., 2014). Another recently proposed function of risk/salience prediction errors is to break habits (Camerer et al., 2024). These behavioral functions can differ across individuals. In line with these notions, pupil diameters were larger when appetitive or aversive outcomes occurred unexpectedly than when the same outcomes occurred expectedly, and pupil diameter correlated with subjectively defined risk/salience prediction errors.

In line with generic surprise processing, objective risk prediction errors commonly increased pupil dilation (as well as insula activity). These findings converge with previous reports of pupil responses correlating with surprise, operationalized as objective risk prediction errors (Preuschoff et al., 2011) and predicting the impact of new information on subsequent learning (Nassar et al., 2012). We advance this literature by showing pupil responses not only to objective but also to subjective risk prediction errors and by revealing that these responses occur preferentially in the aversive domain when risk prediction errors are defined subjectively but not when they are defined objectively.

In contrast to objective risk prediction error processing, pupil dilation and anterior insula activity diverged somewhat for subjective risk prediction errors. For example, while pupil dilation encoded subjective risk prediction errors to outcomes primarily in the aversive domain, anterior insula activation was domain-general, encoding subjective risk prediction errors in both appetitive and aversive domains. This divergence may reflect distinct functions, such as a distinction between autonomic arousal and cognitive expectancy violations (Sara and Bouret, 2012). Pupil-linked arousal responses are thought to partially index the activity of the noradrenergic system (Aston-Jones and Cohen, 2005; Joshi et al., 2016; Lauffs et al., 2020). By extension, our data tentatively suggest that the locus ceruleus may encode subjective risk prediction errors preferentially in the aversive domain. In contrast, to underpin a more domain-general encoding of subjective risk prediction errors (Fouragnan et al., 2017; Haufler et al., 2022), the anterior insula may receive and process also inputs from regions that encode subjective risk prediction errors preferentially in the appetitive domain. Moreover, taking the pupil responses together with the objective risk prediction error signal in the mid-insula (Fig. 3), future neurophysiological or neuropharmacological work may want to test whether the mid-insula more closely tracks norepinephrinergic inputs than the anterior insula.

One limitation worth noting is that our cue phase was not very long (4 s) and the cue–outcome jittering was limited (1.1–4.2 s). Prior work demonstrated that risk anticipation signals build over longer temporal windows in the insula (Preuschoff et al., 2008) and our event timings provided limited capacity to observe such sustained risk anticipation signals. Nevertheless, they revealed phasic risk prediction error signals, which reinforce the role of the anterior insula in learning to anticipate risk.

### Preferential encoding of subjective risk prediction errors

A unique feature of our task is that it required participants to evaluate both cues and outcomes in every trial. This allowed us to directly compute subjective risk prediction errors and dissociate them from objective risk prediction errors. Activity in the anterior insula (and the anterior cingulate cortex) more strongly reflected subjective than objective risk prediction errors. This finding is compatible with the proposal that particularly the anterior insula plays a central role in awareness of internal states and in processing feelings, i.e., the subjective aspect of emotions (Craig, 2009). Anatomically, the anterior insula (along with the anterior cingulate cortex) is special in that layer 5 contains large spindle-shaped (von Economo) neurons, which appear to be unique for higher-order animals (Allman et al., 2011). These neurons, and the regions containing them, have been associated with awareness (Fischer et al., 2016; Cabeen et al., 2023). Together with the preferential coding of objective risk prediction errors by the mid-insula, our findings point to a posterior-to-anterior gradient, extending anteriorly into the adjacent prefrontal cortex reflecting the extent to which aspects of risky and emotionally relevant events are subjectively reportable.

Furthermore, the anterior insula processed subjective risk prediction errors also for neutral liquids and in situations never followed by any liquid (*p* = 0), which may explain the better fit with subjective than objective prediction error regressors. This finding extends previous reports of the insula processing prediction errors for motivationally charged outcomes and events (Preuschoff et al., 2008; Hein et al., 2016; Man et al., 2024) to outcomes and events that bear individual meaning (defined as absolute value). Moreover, the continued encoding of small deviations from neutral predictions suggests that principles of adaptive coding may be at play not only in the domain of signed value prediction errors [e.g., dopamine neurons (Tobler et al., 2005); insula activity (Kirschner et al., 2018)] but also in the domain of unsigned value prediction errors. Through such adaptive encoding, the insula may achieve optimal sensitivity to the most likely motivationally relevant deviations from current predictions in any domain.

### Domain-general and domain-preferential encoding of risk prediction errors

At the time of the outcomes, the anterior insula processed subjective risk prediction errors in a domain-general fashion, whereas at the time of cues, it preferentially did so in the aversive domain. This finding provides an explanation for the existence of two apparently conflicting lines of research. On the one hand, it confirms risk/salience prediction error coding by the anterior insula (Rutledge et al., 2010; Metereau and Dreher, 2013; Fouragnan et al., 2018; Corlett et al., 2022; Haufler et al., 2022; Horing and Büchel, 2022), which is thought to be domain-general by definition. On the other hand, it corroborates the increased sensitivity of the anterior insula for the aversive domain (Kuhnen and Knutson, 2005; Bossaerts, 2010; Čeko et al., 2022) and specifies it for the prediction of future outcomes. More specifically, at cue onset, the anterior insula was significantly more responsive to risk prediction errors occurring in the aversive than in the appetitive domain. This suggests facilitated expectation formation in the aversive domain, reinforcing attentional biases toward negative stimuli (Lauffs et al., 2020). Taken together, domain generality versus specificity of risk processing in the anterior insula appears to depend on event time.

The laterality of anterior insula activation further differentiates these functions. Prior research reported right anterior insula dominance for aversive salience (Simmons et al., 2006; Linnman et al., 2013; Liljeholm et al., 2014), whereas studies of learning-related risk prediction errors in anterior insula often found bilateral or left-lateralized activation (Preuschoff et al., 2008; Metereau and Dreher, 2013). While our finding of left anterior insula engagement during outcome processing is compatible with this distinction, it should not be overinterpreted as direct statistical comparisons did not reveal significant laterality differences.

In future work, it may be interesting to test whether the proposed gatekeeping function of the anterior insula for goal-directed action (Molnar-Szakacs and Uddin, 2022) also is preferentially sensitive to the aversive domain and whether habits are more readily broken by cues predicting outcomes in the aversive domain. The anterior insula appeared to update cue-related predictions of motivationally relevant outcomes more readily with preceding outcome-induced risk prediction errors occurring in the aversive compared with the appetitive domain. Such selective updating can explain the higher sensitivity of the anterior insula for the aversive than the appetitive domain. Note that these biased updating processes concern unsigned prediction errors. Thereby, they seem to complement biased updating with signed prediction errors (Garrett and Sharot, 2017; Kuzmanovic et al., 2018), where people preferentially update their beliefs in response to outcomes that are better than expected, resulting in optimism for signed value. In contrast, the anterior insula appears to show pessimism bias for future unsigned value and maintained encoding of past salient outcomes that occurred in the appetitive domain.

Domain-specific sensitivity to subjective risk prediction errors may also have clinical relevance. Prior research reported increased anterior insula responses to cues predicting aversive outcomes in anxiety-prone individuals (Simmons et al., 2011) and reduced mid-insula activation to shock in individuals with schizophrenia (Linnman et al., 2013). Thus, insular dysfunction in risk prediction error processing may contribute to maladaptive processing of aversive events. By demonstrating a dissociation between cue-related (aversive) and previous outcome-related (appetitive) risk prediction errors at cue onset, our findings suggest that the anterior insula may employ domain-specific time horizons, which provides a basis for better understanding clinically relevant changes in processing salient events.

## Conclusions

We show that errors in the prediction of subjective salience can be made more precise (by mathematically defining salience as risk) and experimentally tractable in a straightforward manner. Importantly, the primary hubs of the salience network (anterior insula and anterior cingulate cortex) preferentially encode subjective rather than objective risk prediction errors and do so at each moment in time. Moreover, the anterior insula updates risk predictions more strongly in the aversive than the appetitive domain, resulting in aversive domain-preferential risk prediction error signals at the time of cues. These findings reconcile two hitherto apparently conflicting research lines (common risk/salience coding in the appetitive and aversive domains versus preferential coding of the aversive domain).
